# Computational imaging with low-order OAM beams at microwave frequencies

**DOI:** 10.1038/s41598-020-68586-y

**Published:** 2020-07-15

**Authors:** Kang Liu, Yongqiang Cheng, Hongyan Liu, Hongqiang Wang

**Affiliations:** College of Electronic Science and Technology, National University of Defense Technology, Changsha, 410073 China

**Keywords:** Engineering, Physics

## Abstract

With the distinguished wavefront characteristics of vortex electromagnetic wave carrying orbital angular momentum (OAM), the OAM beams have been exploited for radar imaging in recent years. In this paper, the computational imaging model is built using OAM wave, which enables the target reconstruction with limited measurements. The measurement matrix is designed, and the target reconstruction method is proposed in the Cartesian coordinate. Simulation results indicate that the proposed computational imaging approach is robust against noise influence. Furthermore, the outdoor experiments are carried out, for the first time, to validate the super-resolution imaging ability of this novel technique. Experimental results show good agreement with theoretical analyses. This work can advance the development of OAM-based sensing technology.

## Introduction

Orbital angular momentum (OAM), as an extrinsic component of the angular momentum, is associated with the wavefront distribution of a beam, which plays an important role in optics and electromagnetics. Generally, the electromagnetic (EM) wave carrying OAM is called vortex EM wave, and has been found great value in wireless communications^1,2^ and radar realms^3^. Especially, vortex EM waves have attracted considerable attention in object detection, imaging, and rotational Doppler sensing, due to the OAM’s degree of freedom, in recent years^4–8^.

In 2013, the OAM beam was firstly introduced into radar target imaging^9^. Afterwards, a rapid development of OAM-based radar technique emerged. Several techniques were developed to generate OAM beams, namely, structure-shaped antenna, antenna array, metalens, and metasurface^10–15^. For OAM-based target imaging, the antenna array was most often used due to that multiple OAM modes can be generated simultaneously. In 2015, the imaging mathematical models for multiple-in-multiple-out and multiple-in-single-out were built in Ref.^3^. Over the past years, several kinds of imaging algorithms have been developed, i.e., back projection (BP), power spectrum density (PSD) estimation, and spatial spectrum estimation^3,16^. In 2017, the imaging principle of OAM-based target imaging was validated by the measurement in an anechoic chamber^17^. The imaging resolution was analyzed and results indicated that the azimuthal resolution is decided by the range of OAM modes used in the detection process.

For the existed OAM-based imaging methods listed above, the high resolution in the azimuthal dimension is usually based on large OAM modes^3,5^, which is much costly for practical radar systems. Hitherto, the computational imaging technique has been widely applied at microwave frequencies to reconstruct the target^18,19^, which can considerably reduce the set of measurements. Since different OAM eigenvalues are orthogonal, this provides the potential for target reconstruction with limited measurements. Therefore, the microwave computational imaging method is developed to achieve high-resolution target profiles with low-order OAM beams, in this paper. Moreover, to the best of the authors’ knowledge, there are no publications reporting the outdoor experiments about OAM-based target imaging.

## Results

### Imaging model

**Figure 1:**
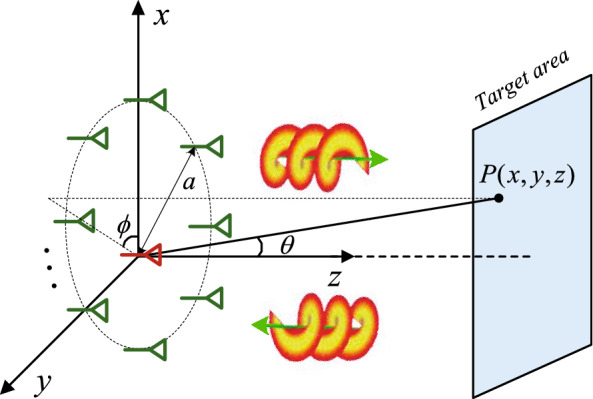
The schematic of target imaging using vortex EM wave. This figure was created by the Microsoft Visio software with the version 2010.

The proposed imaging scheme is presented in Fig. 1, where a uniform circular array (UCA) is exploited to generate vortex EM wave to illuminate the target area. One antenna located at the origin point is used to receive the target echo.

Assuming that the step frequency signal is transmitted by each antenna, based on the OAM-generating method using UCA^3^, the transmitted signal $s(t,l)$ by the UCA takes the formula

1 $$ s(t,l) = \sum\limits_{d = 0}^{D - 1} {u(t - dT_{r} )\sum\limits_{n = 1}^{N} {e^{{i2\pi (f_{0} + d\Delta f)t}} \cdot e^{{il\phi_{n} }} } } $$

where $u(t - dT_{r} )$ denotes the rectangular pulse function, and $T_{r}$ is the pulse repetition interval. $f_{0}$ is the signal frequency of the first pulse, and $\Delta f$ is the step frequency. $\phi_{n}$ indicates the azimuthal position of the $n{\text{th}}$ antenna, $D$ is the number of pulses, and $N$ is the number of antennas. $l$ denotes the OAM index.

Without loss of generality, the target is assumed to be located at the position $P(x,y,z)$, and the echo $s_{r} (t,l)$ can be given by

2 $$ s_{r} (t,l) = \sum\limits_{d = 0}^{D - 1} {u(t - dT_{r} - \tau ) \cdot \sigma \sum\limits_{n = 1}^{N} {e^{{i2\pi (f_{0} + d\Delta f)(t - \tau )}} \cdot e^{{il\phi_{n} }} } } $$

where $\tau = 2\sqrt {(x - a\cos \phi_{n} )^{2} + (y - a\sin \phi_{n} )^{2} } {/}c$ is the time delay, and $\phi_{n} = 2\pi (n - 1)/N$, $n = 1,2, \ldots ,N$. $a$ is the radius of the UCA, and $\sigma$ stands for the target scattering coefficient.

When multiplying Eq. (2) by the reference term $\exp ( - i2\pi f_{0} t)$, it leads to

3 $$ \begin{aligned} s_{r} (t,l) & = \sum\limits_{d = 0}^{D - 1} {u(t - dT_{r} - \tau ) \cdot \sigma \sum\limits_{n = 1}^{N} {e^{{i2\pi (f_{0} + d\Delta f)(t - \tau )}} \cdot e^{{il\phi_{n} }} } } \cdot e^{{ - i2\pi f_{0} t}} \\ & = \sum\limits_{d = 0}^{D - 1} {u(t - dT_{r} - \tau ) \cdot \sigma \sum\limits_{n = 1}^{N} {e^{{i2\pi [d\Delta f(t - \tau ) - f_{0} \tau ]}} \cdot e^{{il\phi_{n} }} } } . \\ \end{aligned} $$

To reconstruct the target, the computational imaging equation is written as

4 $$ {\varvec{S}}_{r} = {\varvec{S}} \cdot {\varvec{\sigma}} + {\varvec{n}} $$

where ${\varvec{S}}_{r}$ is the echo vector, ${\varvec{S}}$ is the measurement matrix, and ${\varvec{\sigma}}$ is the unknown scattering coefficient vector. ${\varvec{n}}$ indicates the unknown noise vector.

Based on Eqs. (3) and (4), the computational imaging equation can be rewritten as follows

5 $$ \left[ {\begin{array}{*{20}c} {s_{r} (t_{1} ,l_{1} )} \\ {s_{r} (t_{2} ,l_{1} )} \\ \vdots \\ {s_{r} (t_{P} ,l_{L} )} \\ \end{array} } \right] = \left[ {\begin{array}{*{20}c} {s^{1} (t_{1} ,l_{1} )} & {s^{2} (t_{1} ,l_{1} )} & \ldots & {s^{Q} (t_{1} ,l_{1} )} \\ {s^{1} (t_{2} ,l_{1} )} & {s^{2} (t_{2} ,l_{1} )} & \ldots & {s^{Q} (t_{2} ,l_{1} )} \\ \vdots & \vdots & \ddots & \vdots \\ {s^{1} (t_{P} ,l_{L} )} & {s^{2} (t_{P} ,l_{L} )} & \ldots & {s^{Q} (t_{P} ,l_{L} )} \\ \end{array} } \right] \cdot \left[ {\begin{array}{*{20}c} {\sigma_{1} } \\ {\sigma_{2} } \\ \vdots \\ {\sigma_{Q} } \\ \end{array} } \right] + \left[ {\begin{array}{*{20}c} {n_{1} } \\ {n_{2} } \\ \vdots \\ {n_{PL} } \\ \end{array} } \right] $$

where the target area is discretized into $Q$ grids, and the target scatters are assumed to be located at the center of each cell. $P$ is the sampling points of the echo in the time domain, and $L$ is the total number of OAM modes used in the imaging. $\sigma_{i} ,\;i = 1,2, \ldots ,Q$ denotes the scattering coefficient of the $i{\text{th}}$ grid.

Though the OAM eigenvalues are orthogonal to each other, the rows of the measurement matrix are coherent since the step frequency signal is used. Thus, Eq. (5) is usually an ill-posed inverse problem, i.e., the number of parameters to be solved is more than the measured information. The stable solutions cannot be obtained by the conventional least-square method^20^.

Generally, the matched filtering approach can solve the unstable phenomenon caused by the ill-posed problem of the imaging equation. And the estimation $\hat{\user2{\sigma }}$ for the target scattering coefficients can be expressed as

6 $$ \user2{\hat{\sigma } = S}^{H} {\varvec{S}}_{r} . $$

The method in Eq. (6) is robust against the noise, however, the main lobes of the reconstructed profiles usually widen and the side lobes always exist. For radar imaging applications, the sparse characteristics of the imaging scene are usually assumed to be the prior information, which can be exploited to reduce the solution instability of the imaging equation and improve the target reconstruction performance. This can be presented as an optimization process

7 $$ \hat{\user2{\sigma }} = \arg \mathop {\min }\limits_{{\varvec{\sigma}}} \left\{ {\left\| {{\varvec{S}}_{r} - {\varvec{S}} \cdot {\varvec{\sigma}}} \right\|_{2}^{2} + \gamma \left\| {\varvec{\sigma}} \right\|_{1} } \right\} $$

where $\gamma$ is a scale weighting coefficient.

### Simulated results

To show the effectiveness of the proposed computational imaging scheme, simulations are carried out and the SBL-based method is used to reconstruct the target. The key scene parameters are listed in Table 1. The size of the imaging cell is set as 1 m and the imaging area is $10\;{\text{m}} \times 10\;{\text{m}}$. The position of the scenario center is denoted as $(5\;{\rm m},500\;{\rm m}, - 10\;{\text{m}})$.

**Table 1:** Key scene parameters.

Parameter	Symbol	Value
Number of antennas	$N$	8
Radius of UCA	$a$	0.4 m
Position of the receiving antenna	$P_{R}$	$( - 2\;{\text{m}},0\;{\text{m}},0\;{\text{m}})$
Signal frequency	$f$	$[9.4\;{\rm GHz},\;9.6\;{\rm GHz}]$
Number of pulses	$N_{pulse}$	20
Sampling rate	$f_{s}$	1.6 GHz

**Figure 2:**
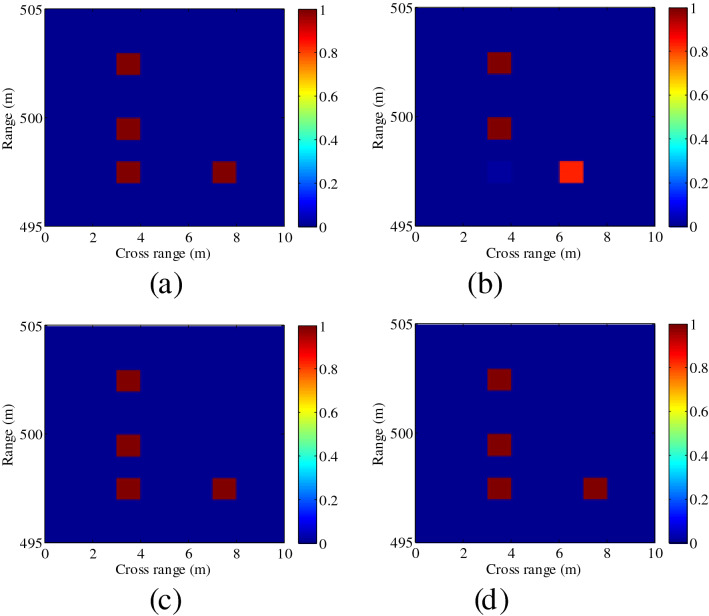
Comparison of the reconstructed results using plane wave and OAM beams. (**a**) Ground truth, (**b**) Plane wave, (**c**) OAM beam *l* = 1, (**d**) OAM beam *l* = 2. The color bar indicates the normalized amplitude. This figure was created by the MATLAB software with the version R2012b.

**Figure 3:**
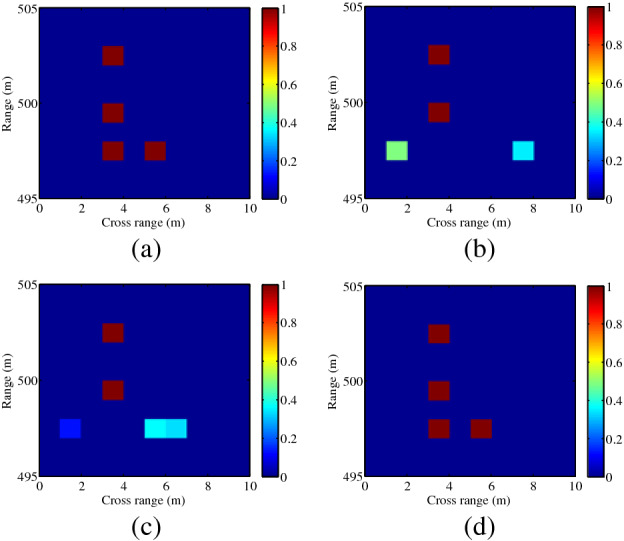
Reconstructed results with different OAM modes. (**a**) Ground truth, (**b**) *l* = 1, (**c**) $l \in [ - 1,1]$, (**d**) $l \in [ - 2,2]$. This figure was created by the MATLAB software with the version R2012b.

The comparison of the target reconstructed results using plane wave and OAM beams is shown in Fig. 2. Results indicate that two targets in the same range cannot be correctly reconstructed in the cross-range dimension when the plane wave is transmitted. In contrast, the helical wavefront of OAM beams can enhance the imaging resolution. Furthermore, two targets are placed closer to each other in the same cross range, and the reconstructed results with different OAM modes are shown in Fig. 3. It is clear from Fig. 3 that more OAM modes are expected to be used to achieve higher resolution.

**Figure 4:**
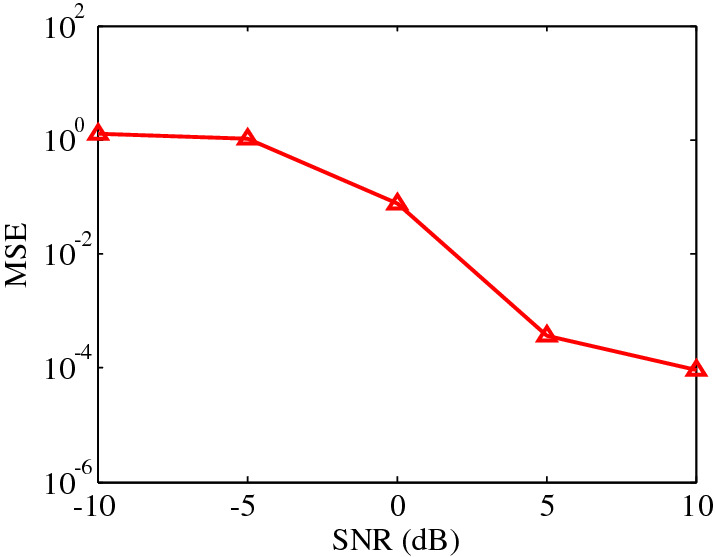
The reconstruction performance as a function of the SNR. This figure was created by the MATLAB software with the version R2012b.

To analyze the noise influence on the imaging performance, the mean square error (MSE)^21^ as a function of the signal-to-noise ratio (SNR) is shown in Fig. 4. In the simulation, the imaging scene is the same as that in Fig. 3, and 100 Monte trials are performed. Results demonstrate that better reconstruction performance can be achieved as the SNR increases.

### Experimental results

**Figure 5:**
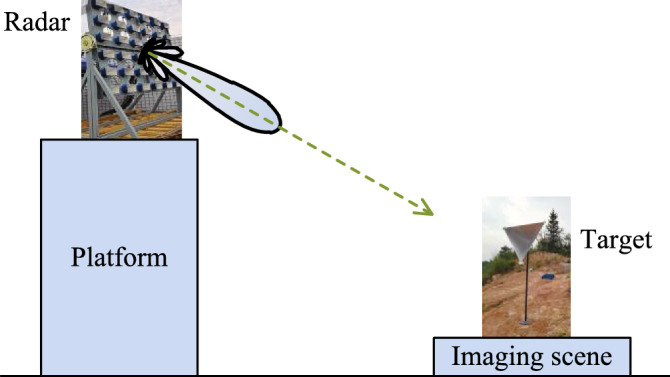
Measurement setup of the outdoor experiments. This figure was created by the Microsoft Visio software with the version 2010.

In this paper, the outdoor experiments are performed, shown in Fig. 5. The array radar placed on a platform is exploited to generate OAM beams. The corner reflectors are placed in the scene for imaging. The main system parameters are listed in Table 1. Considering the outdoor imaging scenario, the noise always exists due to the realistic atmosphere, which will have negative effects on the target reconstruction. To reduce the noise influence, the matched filtering is performed on the echo vector ${\varvec{S}}_{r}$ and the measurement matrix ${\varvec{S}}$, and then Eq. (4) leads to

8 $$ {\varvec{AS}}_{r} = {\varvec{AS}} \cdot {\varvec{\sigma}} + {\varvec{An}} $$

where ${\varvec{A}}$ indicates the matched filtering vector. Since the noise is incoherent, only the signal will be enhanced.

**Figure 6:**
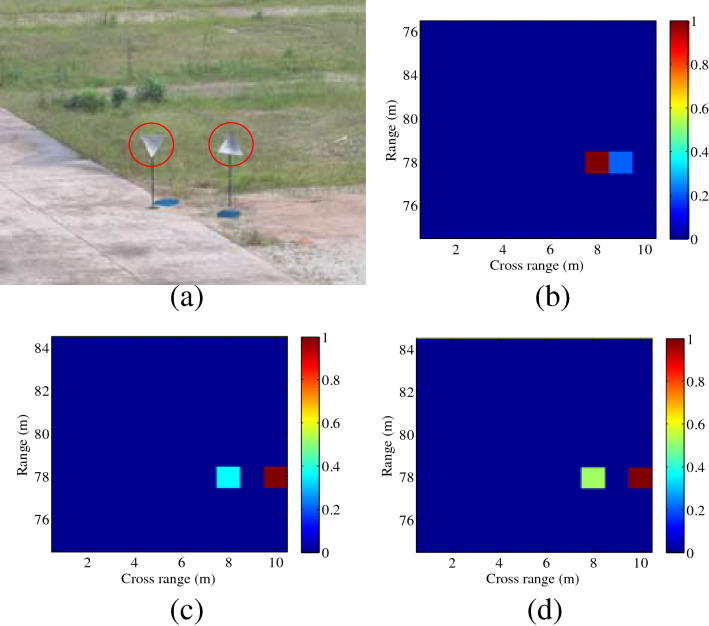
Experimental imaging results with the distance of 79 m (*l* = 2). (**a**) Ground truth, (**b**) plane wave using SBL-based method, (**c**) OAM beam using SBL-based method, (**d**) OAM beam using OMP-based method. This figure was created by the MATLAB software with the version R2012b.

The distance between radar and the scene center is set as 79 m, firstly. Two corner reflectors are placed in the same range, and the distance is 2 m in the cross-range domain. The SBL and OMP methods are used to reconstruct the targets, shown in Fig. 6. Compared with the result using plane waves in Fig. 6b, higher resolution can be achieved by transmitting OAM beams, shown in Fig. 6c,d. It can be seen from Fig. 6 that the spatial cross-range resolution for OAM-based imaging is about $\rho_{a} = 2\;{\text{m}}$, whereas the resolution for conventional imaging is decided by the aperture $\rho_{a} = R \cdot \lambda /(2a)$, i.e., $78 \cdot \lambda /(2a) \approx 3\;{\text{m}}$, where $R$ denotes the target’s position in the range domain, $\lambda$ is the signal wavelength corresponding to the center frequency ($f_{c} = 9.5\;{\text{GHz}}$) of the transmitted signal. Thus, the target cannot be reconstructed using plane waves, shown in Fig. 6b. Further, due to the distinguished positions and the manufacture error of the two corner reflectors, the backward scattering coefficients are usually different, and thus the color scales in Fig. 6c,d are different.

**Figure 7:**
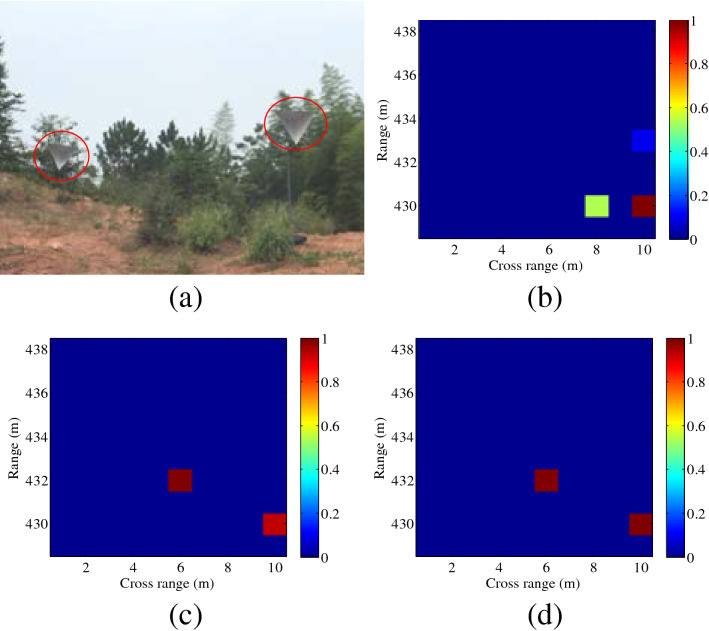
Experimental imaging results with the distance of 433 m (different range values, *l* = 2). (**a**) Ground truth, (**b**) plane wave, (**c**) OAM beam using SBL-based method, (**d**) OAM beam using OMP-based method. This figure was created by the MATLAB software with the version R2012b.

**Figure 8:**
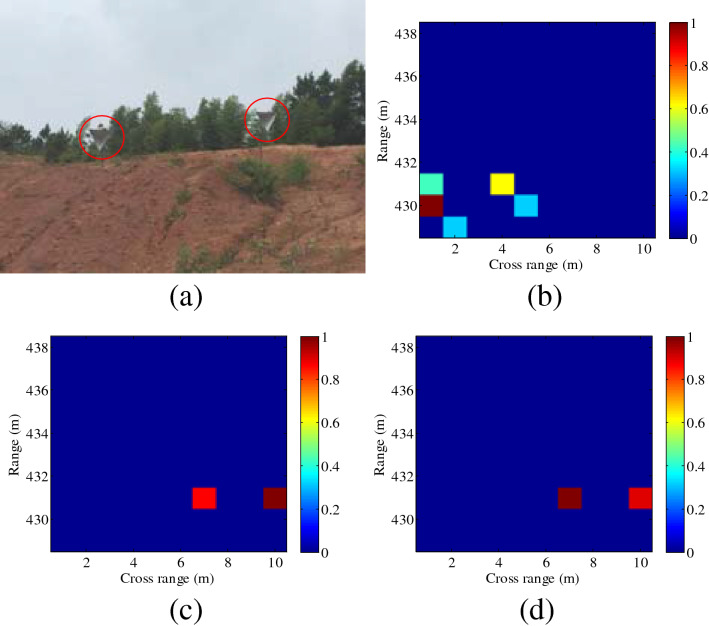
Experimental imaging results with the distance of 433 m (the same range value, *l* = 2). (**a**) Ground truth, (**b**) plane wave using SBL-based method, (**c**) OAM beam using SBL-based method, (**d**) OAM beam using OMP-based method. This figure was created by the MATLAB software with the version R2012b.

Furthermore, the distance between the radar and the scene center is set as 433 m. In Fig. 7, the distances between two corner reflectors are 2 m and 4 m in the range domain and cross-range domain, respectively. In Fig. 8, two corner reflectors are placed in the same range, and the distance is 3 m in the cross-range domain. Results in Figs. 7 and 8 validate the effectiveness of the proposed imaging method, which shows good agreement with the simulation results in Fig. 2. It can be seen from Fig. 8 that the spatial resolution in the cross-range dimension is $\rho_{a} = 3\;{\text{m}}$ , whereas the spatial resolution for plane wave is $\rho_{a} = R \cdot \lambda /(2a) = 431 \cdot \lambda /(2a) \approx 17\;{\text{m}}$. Thus, higher cross-range resolution can be achieved by the proposed imaging method, in this paper.

## Discussion

To conclude, the computational imaging method based on low-order OAM beams has been developed. The imaging model in the Cartesian coordinate was built firstly, and the SBL and OMP approaches were introduced to reconstruct the targets. Outdoor experiments of the OAM-based imaging were carried out, for the first time, to validate the effectiveness of the proposed method. Results indicated that the OAM beam in single mode can be used to reconstruct the target, which can achieve higher resolution than the plane wave. Furthermore, the proposed matched filtering method can enhance the target reconstruction performance for outdoor imaging scene.

This work can be beneficial for the development of OAM in radar imaging. Future work includes the imaging algorithm of extended target and its experimental demonstration.

## Methods

Two different signal processing approaches are employed to reconstruct the target. When the prior information of the samples and the sparse parameters are set, the Bayesian interference can be used to realize the sparse reconstruction and parameter learning, i.e., the sparse Bayesian learning (SBL)^21,22^. Though the columns of the measurement matrix are coherent to each other, the SBL method can still maintain good performance.

Let $h({\varvec{S}}_{r} ;{\varvec{\sigma}})$ be the likelihood function. For an arbitrary probability density function $g({\mathbf{z}})$, it leads to

9 $$ \ln h({\varvec{S}}_{r} ;{\varvec{\sigma}}) = \int {g({\mathbf{z}})\ln h({\varvec{S}}_{r} ;{\varvec{\sigma}})d{\mathbf{z}}} = F(g,{\varvec{\sigma}}) + KL(g||h) $$

where

10 $$ F(g,{\varvec{\sigma}}) = \int {g({\mathbf{z}}){{\rm ln}}\left( {\frac{{h({\varvec{S}}_{r} ,{\mathbf{z}};{\varvec{\sigma}})}}{{g({\mathbf{z}})}}} \right)d{\mathbf{z}}} $$

11 $$ KL(g||h) = - \int {g({\mathbf{z}}){{\rm ln}}\left( {\frac{{h({\mathbf{z|}}{\varvec{S}}_{r} ;{\varvec{\sigma}})}}{{g({\mathbf{z}})}}} \right)d{\mathbf{z}}} $$

where the E-step and M-step are in the following

12 $$ \begin{aligned} & \text{E-step}{:}\;\ln \,g_{j} = \left\langle {\ln h({\varvec{S}}_{r} ,{\mathbf{z}};{\varvec{\sigma}})} \right\rangle_{i \ne j} + const \\ & \text{M-step}{:}\;\;\hat{\user2{\sigma }}^{NEW} = \arg \,\mathop {\max}\limits_{{\varvec{\sigma}}} \,F(g^{NEW} ,{\varvec{\sigma}}). \\ \end{aligned} $$

Furthermore, the orthogonal matching pursuit (OMP)^23^ is also applied to reconstruct the target. Let $\left\{ {{\varvec{x}}_{1} , \ldots ,{\varvec{x}}_{PL} } \right\}$ be a sequence of measurement vectors in ${\mathbb{R}}^{Q}$. The main procedure can be given by

13 $$ \begin{aligned} {\varvec{x}}_{i} &= \arg \mathop {\min }\limits_{{\varvec{x}}} \left\| {{\varvec{S}}_{r} - {\varvec{S}}_{i} {\varvec{x}}} \right\|_{2}^{2} \\ {\varvec{S}}_{i} &= [{\varvec{S}}_{i - 1} ;\;{\varvec{S}}_{{\lambda_{i} }} ] \\ \lambda_{i} &= \arg \max_{q = 1, \ldots ,Q} \left| {({\varvec{S}}^{{\text{T}}} )_{q} \cdot {\varvec{r}}_{i - 1} } \right| \\ \end{aligned} $$

where $i$ denotes the iteration number, and ${\varvec{S}}_{i}$ is the $i{\text{th}}$ row of the measurement matrix ${\varvec{S}}$. ${\varvec{r}}_{i} = {\varvec{S}}_{r} - {\varvec{a}}_{i}$ is the residual, and ${\varvec{a}}_{i} = {\varvec{S}}_{i} {\varvec{x}}_{i}$ is the approximation of the echo vector ${\varvec{S}}_{r}$. The nonzero elements of the estimation $\hat{\user2{\sigma }}$ is recovered from ${\varvec{x}}_{i}$.
